# Recent Advances in Antimicrobial Polymers: A Mini-Review

**DOI:** 10.3390/ijms17091578

**Published:** 2016-09-20

**Authors:** Keng-Shiang Huang, Chih-Hui Yang, Shu-Ling Huang, Cheng-You Chen, Yuan-Yi Lu, Yung-Sheng Lin

**Affiliations:** 1 The School of Chinese Medicine for Post-Baccalaureate, I-Shou University, Kaohsiung 84001, Taiwan; huangks@isu.edu.tw; 2 Department of Biological Science and Technology, I-Shou University, Kaohsiung 84001, Taiwan; chyang@isu.edu.tw; 3 Department of Chemical Engineering, National United University, Miaoli 36003, Taiwan; simone@nuu.edu.tw (S.-L.H.); wayne20410@gmail.com (C.-Y.C.); ilovego0014010@yahoo.com.tw (Y.-Y.L.)

**Keywords:** antimicrobial, polymer, microbe, bacteria, review

## Abstract

Human safety and well-being is threatened by microbes causing numerous infectious diseases resulting in a large number of deaths every year. Despite substantial progress in antimicrobial drugs, many infectious diseases remain difficult to treat. Antimicrobial polymers offer a promising antimicrobial strategy for fighting pathogens and have received considerable attention in both academic and industrial research. This mini-review presents the advances made in antimicrobial polymers since 2013. Antimicrobial mechanisms exhibiting either passive or active action and polymer material types containing bound or leaching antimicrobials are introduced. This article also addresses the applications of these antimicrobial polymers in the medical, food, and textile industries.

## 1. Introduction

Microbes are living organisms, such as bacteria, fungi, and parasites, which are the critical sources of infections [1]. Infectious diseases result from pathogenic microbes and kill more people than any other single cause [2]. An antimicrobial is an agent used to kill microbes or inhibit their growth. Although numerous antimicrobial drugs have been developed to kill or inhibit microbes, many infectious diseases remain difficult to treat [3,4]. Antimicrobial polymers were discovered since 1965 [5] and have attracted considerable attention in both academic and industrial research. Table 1 shows recent review articles on antimicrobial polymers from various perspectives. These reviews focus on methods for producing antimicrobial polymers and various applications of antimicrobials. Increasing efforts develops learning from nature, green or nontoxic biocides. The medical, food, and textile industries are three major areas of applied antimicrobials. More than 27,845 patents for antimicrobial polymers have been filed in the Google Patent Search database since 2013. In addition, antimicrobial medical devices have attracted substantial attention in clinical trials [5]. Compared with their small molecular counterparts, antimicrobial polymers demonstrate superior efficacy, reduced toxicity, minimized environmental problems, and greater resistance [6].

When microbes adhere to a substrate, they excrete biofilms to anchor themselves to the substrate. In biofilms, cells grow in multicellular aggregates and become embedded within a self-produced matrix of an extracellular polymeric substance. A biofilm extracellular polymeric substance is a polymeric conglomeration composed of polysaccharides and other components, such as proteins and DNA. Defective biofilms cannot offer an environment for microbes to grow. Therefore, antimicrobial applications entail strategies for preventing microbial viability or adhesion. For example, antimicrobial peptides act primarily by disrupting the bacterial cell membrane, and heparin exhibits anti-adhesive activity and hydrophilic characteristics that prevent the growth of microbes [21]. Several reviews have described antimicrobial management [22,23,24,25,26,27,28]. Biofilms are difficult to remove and resist many biocides. Therefore, to prevent the spread of diseases, inhibiting biofilm formation and reducing microbial attachment are a more promising antimicrobial strategy than killing microbes [17,29].

Many promising antimicrobial polymers have been reported, and the number of FDA-approved antimicrobial polymers has increased drastically in the past decade [5]. This review describes the new developments in antimicrobial polymers over the past three years. According to the mechanism of antimicrobial activity, the activity of antimicrobial polymers can be categorized as either passive or active (Section 2). Based on the polymer material type, antimicrobial polymers can be classified as bound or leaching antimicrobials (Section 3). These antimicrobial polymers are applied in the medical, food, and textile industries (Section 4). Finally, the conclusion and prospects for future research are addressed (Section 5).

## 2. Passive or Active Action

### 2.1. Passive Action

**Figure 1:**
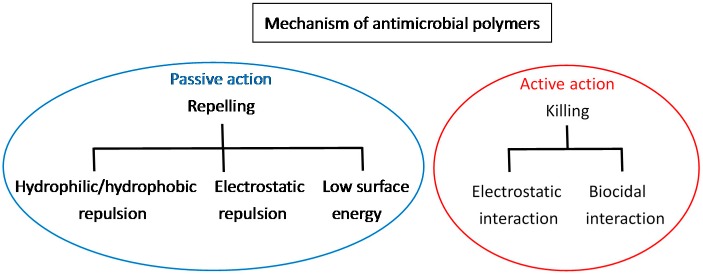
The schematic reaction mechanisms of passive and active action of the antimicrobial polymers.

A passive polymer layer can reduce protein adsorption on its surface, thereby preventing the adhesion of bacteria. However, although passive surfaces repel bacteria, they do not actively interact with or kill bacteria. Due to the mainly hydrophobic and negatively-charged properties of microbes, passive polymers should be either (1) hydrophilic; (2) negatively-charged; or (3) have a low surface free energy (Figure 1) [8,30]. Typical passive polymers comprise (1) self-healing, slippery liquid-infused porous surface (SLIPS), such as poly(dimethyl siloxane); (2) uncharged polymers, such as poly(ethylene glycol) (PEG), poly(2-methyl-2-oxazoline), polypeptoid, polypoly(*n*-vinyl-pyrrolidone), and poly(dimethyl acrylamide); and (3) charged polyampholytes and zwitterionic polymers, such as phosphobetaine, sulfobetaine, and phospholipid polymers [31,32]. Table 2 lists selected passive polymers and their antimicrobial applications. Among these passive polymers, PEG has been extensively studied and has demonstrated excellent antimicrobial effects in drastically reducing protein adsorption and bacterial adhesion. Due to high chain mobility, large exclusion volume, and steric hindrance effect of highly hydrated layer [30], PEG has been the most commonly used passive antimicrobial material [33], and research has shown that it exhibits high antifouling ability to prevent protein and cell adhesion effectively, consequently preventing the growth of microbes.

### 2.2. Active Action

Active polymers actively kill bacteria that adhere to the polymer surface. Polymers functionalized with active agents, such as cationic biocides, antimicrobial peptides, or antibiotics, can kill bacteria on contact. The mechanism of polymers killing microbes depends on the active agents (Figure 1). The most widely used active antimicrobial polymers are functionalized with positively-charged quaternary ammonium, which interacts with the cell wall and destroys the cytoplasmic membrane, resulting in the leakage of intracellular components and consequent cell death [20]. In addition, polyethylenimine, polyguanidine, and *N*-halamine are representative polymers that demonstrate active antimicrobial activity. Polyethylenimine brings about bacterial cell membrane rupture by the electrostatic interaction between polyethylenimine and the cell membrane. Polyguanidine has bacterial growth inhibition through adhesion and subsequent disruption of Ca^2+^ salt bridges or cell death. *N*-halamine makes cell inhibition or inactivation by action of the oxidative halogen targeted at thio or amino groups of cell receptors [6]. Table 3 lists active polymers and their antimicrobial applications, indicating that most of these new antimicrobials materials are based on quaternary ammonium salts.

## 3. Bound or Leaching Antimicrobials

Several recent comprehensive state-of-the-art reviews summarize the progress of and research on antimicrobial polymers [6,11]. Antimicrobial polymers can be divided into three types: polymeric biocides, biocidal polymers, and biocide-releasing polymers [5]. Recently, synergistic combination has been commonly used to provide multiple functional antimicrobials for fighting pathogens.

### 3.1. Polymeric Biocides

Polymeric biocides are polymers that covalently link bioactive repeating units with antimicrobial activity such as amino, carboxyl, or hydroxyl groups [8,14,18]. The polymerization process may either enhance or reduce the antimicrobial activity of bioactive functional groups. Table 4 lists examples of polymeric biocides synthesized from antimicrobial monomers.

### 3.2. Biocidal Polymers

Requiring no bioactive repeating units, the antimicrobial site of biocidal polymers is embodied by the entire macromolecule. Many biocidal polymers contain cationic biocides, such as quaternary ammonium, phosphonium, tertiary sulfonium, and guanidinium. Microbes generally have a negative charge at the outer membrane of the cell. Cationic polymers can lead to the destabilization of the cell surface and the ultimately induction of bacterial death [19]. The antimicrobial activity of cationic polymers relate to the charge density of cationic groups.

Table 5 lists examples of biocidal polymers in relevant literature. Due to its properties of nontoxicity, biodegradability, and biocompatibility, chitosan is the most representative natural material exhibiting inherent antimicrobial activity. The antimicrobial activity of chitosan depends heavily on pH value. With a pH value of less than p*K*_a_, electrostatic interaction occurs between protonated amino groups and the cell wall. When the pH is higher than the p*K*_a_ value, the antimicrobial activity of chitosan derives from hydrophobic interaction and chelation effects. Other natural antimicrobial polymers include heparin, poly-ε-lysine, and gramicidin A [6]. Antimicrobial peptides have been recognized as promising candidates for the new generation of antibacterial surfaces [10,58,59]. Thus far, more than 1000 antimicrobial peptides have been found. In addition to directly killing microbes by disrupting cell membranes and inhibiting cellular processes, the antimicrobial mechanisms of peptides can also exert immunomodulatory effects resulting in microbial clearance through stimulation of the noninflammatory host immune response [9]. The challenges of microbial peptides for therapeutic use include unwanted side effects, high production costs, deficient stability, and adaptive antimicrobial resistance [9].

### 3.3. Biocide-Releasing Polymers

Biocide-releasing polymers can be realized by (1) polymerization of biocide-releasing molecules to polymeric backbone; or (2) polymer/biocide-releasing molecules composites. The polymer in biocide-releasing systems is used as a carrier for biocides. Polymers exhibit antibacterial properties through the incorporation of antibiotic and/or antiseptic compounds. The controlled release system of biocide-release polymers has numerous advantages such as maintaining a high local biocide concentration close to microbes and facilitating the delivery of biocides with short in vivo half-lives. This type of antimicrobial polymer demonstrates great potential for use in the medical industry. Numerous biodegradable polymer devices have been developed as antibiotic carriers for various applications [60,61,62,63]. Table 6 shows various biocide-releasing polymer systems, including new polymer composites that exhibit antimicrobial activity.

A chitosan-agarose hybrid material and nanocomposite ionogels decorated with silver were produced using an ionic liquid, 1-butyl-3-methylimidazolium chloride. The prepared antimicrobial composite ionogels were biocompatible and demonstrated favorable electrical conductivity, as well as thermal and conformational stability [64]. A resorbable antibiotic-eluting polymer composite bone void filler was developed to exhibit both osteoconductive and antimicrobial properties for reducing the rates of orthopedic device-related infections [65]. Synergistic combinations of iron-sequestering polymers and conventional antibiotics may drastically reduce the minimum inhibitory concentrations of antibiotics and offer a promising early intervention or adjuvant to antibiotics [66]. Incorporating crystal violet and di(octyl)­phosphinic-acid-capped zinc oxide nanoparticles into medical-grade silicone can provide a dual-mechanism antimicrobial polymer as a strategy for reducing the risk of infection [67].

In addition, antimicrobial polymers can be classified as surface-bound or solution-based polymers. Surface-bound polymers have direct antimicrobial activity on the polymer surface. However, solution-based polymers need to be used in solutions to have antimicrobial activity. In general, biocidal polymers are surface-bound polymers, while biocide-releasing polymers are solution-based polymers to release biocides in solutions. Depending on the property of bioactive repeating units, polymeric biocides can be surface-bound or solution-based polymers.

## 4. Applications

The major areas of applied antimicrobial polymers are the medical, food, and textile industries. The recent advances in these three areas are addressed as follows.

### 4.1. Medical Industry

The surfaces of all medical devices provide an environment for microbial growth, and are susceptible to microbial infection. Despite continual improvements in materials and techniques, most hospital-acquired infections originate from medical devices. An innovative antimicrobial copolymer of 4-vinyl-*n*-hexylpyridinium bromide (VP) and dimethyl(2-methacryloyloxyethyl) phosphonate (DMMEP) was developed to reduce biofilm formation and to improve the long-term use of medical devices. Coating a copolymer (VP:DMMEP 30:70) on titanium drastically reduces the adhesion of various pathogenic bacteria (e.g., *Streptococcus sanguinis*, *Escherichia coli*, *Staphylococcus aureus*, *Staphylococcus epidermidis*). Furthermore, soft tissue cells (human gingival or dermis fibroblasts) are minimally affected by such a coating [76].

Antimicrobial peptides and synthetic mimics of antimicrobial peptides are a new generation of antimicrobial agents with high antimicrobial, broad spectrum activity against a variety of pathogens and modulation of the immune response [77,78,79,80,81,82]. Antimicrobial wound-dressings composed of cotton gauze containing antimicrobial peptides incorporated with polycation (chitosan) and polyanion (alginic acid sodium salt) exert a high antimicrobial effect (in the range of 4–6 log reduction) for *Staphylococcus aureus* and *Klebsiella pneumonia*. These dressings have also been proven to be noncytotoxic to normal human dermal fibroblasts [83].

A novel controlled release zinc oxide/gentamicin-chitosan composite gel was developed. By slowly releasing the antibiotic, this composite gel demonstrated highly effective antimicrobial properties, inhibiting *Staphylococcus aureus* and *Pseudomonas aeruginosa* growth under both planktonic and surface-attached conditions. When used in a wound dressing, it maintained a moist environment at the wound interface and provided a cooling sensation and soothing effect. Moreover, this system is fully scalable to any other soluble drug because the entire solution remains trapped in the ZnO-chitosan composite gel [84].

The infection of catheter-associated urinary tract is the commonest hospital-acquired infection. Impregnation of urinary catheters with a combination of rifampicin, sparfloxacin and triclosan was developed. The release of the drugs from the silicone catheter segments were more than one month. The antimicrobial catheters could prevent colonization of *Proteus mirabilis*, *Staphylococcus aureus*, and *Escherichia coli* for 7–12 weeks. The impregnated catheters might reduce catheter-associated urinary tract infection in both short-term and long-term urinary catheter use [85].

### 4.2. Food Industry

Food safety and quality have attracted increasing attention because of concerns about consumer health. The advent of new technologies has been addressed by the food industry to reduce the risks to consumer health. In particular, substantial progress in food packaging has been achieved using antimicrobial polymers.

Nisin is the only bacteriocin approved as a food preservative because of its favorable properties of negligible toxicity and antibacterial effectiveness [86]. Nisin-loaded chitosan/poly(l-lactic acid) antimicrobial films were developed for applications in food packaging. The diffusion process of antimicrobial nisin from the manufactured film is spontaneous and endothermic. The well-controlled release of nisin from the film demonstrates high antimicrobial activity against *Staphylococcus aureus* [87].

Antimicrobial packing films were developed by compounding low-density polyethylene and its blend of ethylene vinyl acetate with potassium sorbate. A new approach to incorporating preservatives into a polyolefin matrix by using glycerol mono-oleate as a dispersant was reported to obtain uniform dispersions of the preservatives in the packing films, thereby significantly improving the thermal stability with no viscosity reduction [88].

A cellulose acetate film incorporated with a solution of bacteriophages was developed for food packaging. This film showed antimicrobial activity against *Salmonella* Typhimurium ATCC 14028, and the bacteriophages could remain viable for 14 days [89].

### 4.3. Textile Industry

Textiles are favorable substrates for microbial growth under appropriate conditions of temperature and moisture. Antimicrobial agents have yielded new opportunities for additional applications involving textile fibers. The market for antimicrobial textiles has grown dramatically over the past two decades.

Ag:ZnO/chitosan nanocomposite coatings were developed using a modified sol-gel method with 3-glycidyloxypropyltrimethoxysilane and tetraethoxysilane as functionalization agents and were applied to antimicrobial fabrics. This prepared hybrid nanocomposite demonstrated high antimicrobial activity and exhibited higher thermal stability than that of chitosan. Composite coatings on the textile blend of cotton/polyester (50%/50%) exerted the most advanced effect [90].

Natural fibers contributing to the reduction of environmental pollution as well as the burden of waste disposal have recently received considerable attention and become highly valuable materials. A new type of textile consisting of mulberry fibers uniformly laden with titania nanorods was prepared using sol-gel electrospinning and facile dip-coating methods. The mulberry fiber-TiO_2_ composite textile exhibited improved antimicrobial activity compared with pure mulberry textile. Furthermore, the advantages of this unique natural-synthetic composite textile are its anti-yellowing and self-cleaning properties, which are due to the scattering effect of UV radiation by titania nanorods [91].

For fabricating ecofriendly antimicrobial textile material, the impregnation of polypropylene (PP) and corona-modified PP nonwoven material with thymol by super-critical solvent impregnation with carbon dioxide as a working fluid was proposed. The thymol impregnation yield was approximately 7% for both PP and corona-modified PP nonwoven fabrics, providing antimicrobial activity against *S. aureus*, *E. coli*, and *Candida albicans*. Nevertheless, the higher rate of thymol release from the corona-modified material was due to the higher fiber surface hydrophilicity [92].

## 5. Conclusions

Recently, antimicrobial polymers have received considerable attention in both academic and industrial research. This mini-review summarizes the advances made in antimicrobial polymers since 2013. Passive antimicrobial polymers preventing bacterial adhesion and growth provide a more promising strategy than that of killing microbes directly by using active antimicrobial polymers. Among three bound or leaching polymers–polymeric biocides, biocidal polymers, and biocide-releasing polymers–the biocide-releasing system demonstrates the most potential because of the controlled release characteristics. Despite the substantial progress of antimicrobial polymers, the precise mechanisms underlying antimicrobial interaction with microbes necessitates further clarification. In particular, biofilm-associated mechanisms will require an intensive effort to design a promising antimicrobial agent. Combining diverse antimicrobial mechanisms may contribute to a more effective antimicrobial polymer. Further challenges are developments of long-acting or reusable antimicrobial polymers, a broad range of antimicrobial activity, and an activity-controlled system on demand sites.

## Figures and Tables

**Table 1:** Recent review articles of antimicrobial polymers.

Subject	Topic	Reference
Application	Stimuli-responsive polymeric materials for human health applications	[7]
	Antimicrobial polymers for anti-biofilm medical devices	[8]
	Antimicrobial peptides in the treatment of bacterial biofilm infections	[9]
Overview	Antimicrobial peptides and enzymes	[10]
	Anti-infectious surfaces achieved by polymer modification	[11]
	Antimicrobial polymers	[6]
	Antimicrobial polymers with metal nanoparticles	[12]
Synthesis and characteristic	Antimicrobial *N*-halamine polymers and coatings	[13]
	Antimicrobial modifications of polymers	[14]
	Antibacterial dental resin composites	[15]
	Novel formulations for antimicrobial peptides	[16]
	Coatings and surface modifications imparting antimicrobial activity to orthopedic implants	[17]
	Antimicrobial activity of chitosan derivatives containing *n*-quaternized moieties	[18]
	Cationic polymers and their self-assembly for antibacterial applications.	[19]
	Antimicrobial polymeric materials with quaternary ammonium and phosphonium salts	[20]

**Table 2:** Examples of passive polymers for antimicrobial applications.

Polymer	Target	Remark	Reference
Poly(ethylene glycol)	*Staphylococcus aureus*, *Escherichia coli*, *Pseudomonas aeruginosa*	Used as neutral polymer brush systems to prevent protein and cell adhesion	[33]
Poly(sulfobetaine methacrylate)	*Pseudomonas aeruginosa*, *Staphylococcus epidermidis*	Resist protein adsorption, cell attachment, and bacterial adhesion	[34]
Poly[3-dimethyl (methacryloyloxyethyl) ammonium propane sulfonate-*b*-2-(diisopropylamino)ethyl methacrylate]	*Staphylococcus aureus*	Zwitterionic coronae and pH-responsive cores can impart bacterial anti-adhesive properties	[35]
Poly(2-methyl-2-oxazoline)	*Escherichia coli*	Dual-functional antimicrobial surface of poly(l-lysine)-graft-poly(2-methyl-2-oxazoline)-quarternery ammonium	[36]
Albumin, whey	*Bacillus subtilis*, *Escherichia coli*	No bacterial growth was observed on albumin-glycerol and whey-glycerol after 24 h inoculation	[37]
Polyphenols	*Streptococcus mitis*, *Fusobacterium nucleatu*, *Porphyromonas gingivalis*	Effective against periodontal bacteria	[38]

**Table 3:** Examples of active polymers for antimicrobial applications.

Polymer	Target	Antimicrobial Substance	Remark	Reference
Nisin-immobilized organosilicon	*Bacillus subtilis*	Nisin	Superior antimicrobial activity, and resistant to several cleaning conditions	[39]
Polyurethane containing quaternary ammonium	*Staphylococcus aureus*, *Escherichia coli*	Quaternary ammonium	Good antimicrobial activities against even at low concentrations (5 wt %)	[40]
Poly(*n*,*n*-diethylethylendiamine-co-yrosol-based acrylic)	*Staphylococcus epidermidis*, *Staphylococcus aureus*	Tertiary amine	Combination of two active compounds provide a synergistic action against biofilms and suppress reactive species oxygen	[41]
Organosilicon quaternary ammonium chloride	*Staphylococcus aureus*	Quaternary ammonium	Exerted long-lasting antimicrobial activity for at least four hours	[42]
Poly(2-(dimethylamino)ethyl methacrylate) tethering quaternary ammonium	*Bacillus subtilis*, *Escherichia coli*	Quaternary ammonium	Higher C–N^+^ content and relatively smooth morphology would find potential antimicrobial activity	[43]
Acrylamide polymers with quaternary ammonium	*Staphylococcus albus*, *Escherichia coli*, *Rhizoctonia solani*, *Fusarium oxysporum*	Quaternary ammonium	Benzyl group attached to nitrogen atom showed better inhibitory effect on bacteria and phytopathogenic fungi	[44]

**Table 4:** Examples of polymeric biocides for antimicrobial applications.

Monomer	Target	Antimicrobial Substance	Remark	Reference
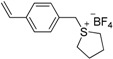	*Staphylococcus aureus*, *Escherichia coli*	Sulfonium salt	A high antibacterial activity against Gram-positive bacteria than Gram-negative bacteria	[5]
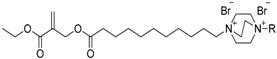	*Staphylococcus aureus*, *Escherichia coli*	Quaternary ammonium	Activity depends on the length of hydrophobic segments	[20]
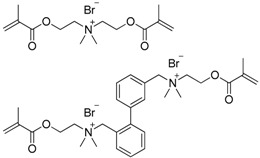	*Escherichia coli*	Quaternary Ammonium	Antimicrobial dental materials	[20]
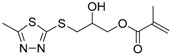	*Micrococcus luteus*, *Staphylococcus aureus*, *Bacillus subtilis*	Benzimidazole	Against Gram-positive bacterial strains MIC values 5.4–53.9 μM	[45]
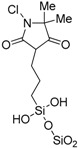	*Staphylococcus aureus*, *Escherichia coli*	Halogen	Inactivate 100% *Staphylococcus aureus* and *Escherichia coli* with a contact time of 10 and 30 min	[46]
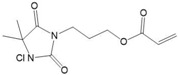	*Staphylococcus aureus*, *Escherichia coli*	*N*-halamine	Excellent biocidal efficacy by inactivating 100% of the bacteria with the contact times less than 10 min	[47]

**Table 5:** Examples of biocidal polymers for antimicrobial applications.

Polymer	Target	Remark	Reference
Quaternary ammonium polyethyleneimine	Gram-positive and Gram-negative bacteria	*n*-alkylated polyethyleneimine has effective antimicrobial activity, dependent on the hydrophobic and positively charged immobilized long polymeric chains	[15]
Quaternary phosphonium modified epoxidized natural rubber	*Staphylococcus aureus*, *Escherichia coli*	Moderate growth inhibition of microbes	[48]
Arginine–tryptophan-rich peptide	Gram-positive and Gram-negative bacteria	Retain antimicrobial functionality for at least 21 days, showing negligible cytotoxicity	[49,50]
Guanylated polymethacrylate	*Staphylococcus epidermidis*, *Candida albicans*	Guanidine copolymers were much more active compared to the amine analogues	[51]
Chitosan	Bacteria, yeast, fungi	Widely-used antimicrobial agent either alone or blended with other compounds	[52,53,54]
Ammonium ethyl methacrylate homopolymers	Methicillin-resistant *Staphylococcus aureus*, *Escherichia coli*	Very little or no hemolytic activity and higher inhibitory effects against Gram-positive bacteria than Gram-negative bacteria	[55]
Metallo-terpyridine carboxymethyl cellulose	*Staphylococcus aureus*, *Streptococcus thermophilus*, *Escherichia coli*, *Saccharomyces cervisiae*	Minimum inhibitory concentration ranged from 6 to 8 mg/L to achieve ≥90% inhibition	[56]
Poly(*n*-vinylimidazole) modified silicone rubber	*Pseudomonas aeruginosa*, *Staphylococcus aureus*	More antibacterial activity against *Pseudomonas aeruginosa* than *Staphylococcus aureus*	[57]

**Table 6:** Examples of biocide-releasing polymers for antimicrobial applications.

Polymer	Target	Antimicrobial Substances	Remark	Reference
Dextrans	*Staphylococcus aureus*	Gentamicin	Enhance gentamicin stability over time and prolong drug release for six days	[60]
Poly-l-lysine, polyethylene glycol	*Staphylococcus aureus*	Staphylolytic LysK enzyme	LysK can lyse bacteria	[68]
Poly(octanediol-co-citrate)	*Staphylococcus aureus*, *Escherichia coli*	Choline chloride, tetraethylammonium bromide, hexadecyltrimethylammonium bromide, methyltriphenylphosphonium bromide	Preserve cytocompatibility while showing elastic properties advantageous for wound dressings	[69]
Cyclodextrin	*Staphylococcus aureus*, *Escherichia coli*	Triclosan	Reduce drug amount to inhibit pathogen growth and toxic impact on environmental strains	[70]
Poly(methyl methacrylate)	*Pseudomonas aeruginosa*, *Staphylococcus aureus*	Silver	Light-activated antimicrobial materials doped with porphyrin and sliver	[71]
Poly(methyl methacrylate)	*Staphylococcus epidermidis*, *Escherichia coli*	Silver, nanoparticles, and imidazole complex	Time-dependent antimicrobial activities	[72]
Cyclodextrin	*Staphylococcus aureus*, *Escherichia coli*	Silver, chitosan	Cyclodextrin stabilized Ag-chitosan and provided higher antimicrobial activity	[73]
Acrylic bone cements	*Enterococcus faecalis* V583	Chlorhexidrina	Retain both mechanical and antimicrobial properties	[74]
Polycaprolactone	*Staphylococcus aureus*, *Pseudomonas aeruginosa*	Silver	A strong antimicrobial and anti-biofilm properties	[75]
